# An In-Depth Examination of Perceptions of Physical Activity in Regularly Active and Insufficiently Active Older African American Women: A Participatory Approach

**DOI:** 10.1371/journal.pone.0142703

**Published:** 2015-11-10

**Authors:** Emerson Sebastião, Wojtek Chodzko-Zajko, Andiara Schwingel

**Affiliations:** Department of Kinesiology and Community Health, University of Illinois at Urbana-Champaign, Illinois, United States of America; Texas A&M University, UNITED STATES

## Abstract

Despite considerable research and programmatic efforts to alleviate racial/ethnic disparities in physical activity (PA), disparities in PA among older minorities and major racial ethnic groups persist. This study explored perceptions of PA among regularly active (RA) and insufficiently active (IA) older African American women (AAW) and the factors that influence (positively and negatively) their physical participation in their socio-cultural environment. A total of 20 AAW aged 60 to 80 years participated in a cross-sectional mixed-methods study (i.e., qualitative and quantitative) employing participatory research approaches (i.e., photoelicitation) along with an objective assessment of PA. Nine women were considered RA and 11 IA according to current PA recommendations. RA and IA women held two major beliefs about the nature of PA (i.e., PA as a broadly defined construct that goes beyond traditional exercise routines; and PA and exercise are synonymous and can be used interchangeably) and had a good understanding of its benefits. Participants in both groups did not know about the importance of PA intensity for health benefits. Barriers and facilitator of PA were found to be similar among RA and IA participants. Special attention should be paid to providing access to no or low cost opportunities for PA participation in safe environments.

## Introduction

The numerous physical and psychological benefits of physical activity (PA) can be achieved by people of all ages and ethnic/racial backgrounds [1]. African Americans are disproportionally affected by many chronic conditions (e.g., diabetes, cardiovascular disease, some types of cancers, and disabilities) when compared with other racial groups [2,3]. Engaging in recommended amounts of PA has the potential to minimize the physiological effects of an otherwise sedentary and inactive lifestyle and increase life expectancy and healthy living by preventing or delaying the onset and progression of chronic diseases and disabling conditions [4,5]. Despite its well-known benefits, rates of PA participation at recommended levels are low in the African American population [6], and there is evidence that older African American women (AAW) are among the least active members of society [7].

The promotion of a physically active lifestyle as an affordable and effective means to prevent and treat chronic disease and to improve quality of life and well-being has become a priority for many researchers, government agencies, policy makers and health professionals. However, despite considerable efforts to alleviate racial/ethnic disparities in PA, disparities persist [8,9]. Efforts have been made to assess and understand perceptions and attitudes influencing PA participation among minorities and underserved groups [10–12]. For example, one study examined perceptions, barriers, and facilitators to PA and exercise among seven underserved and ethnically diverse older adults employing a focus group design. The authors observed that participants, in general, viewed PA and exercise as mean to promote health; and identified both health and social benefits related to being physically active. Furthermore, participants also expressed the notion that PA programs should provide culturally-appropriate activities, foster social relationships among participants, and partner with social service agencies to provide programming at convenient times and in convenient and accessible locations. Mathews et al. [11] observed that common barriers to PA among different ethnic groups aged 50 years and older include health problems, fear of falling, and inconvenience. Common facilitators included positive outcome expectations, social support, and program access.

Although studies can be found that explore factors influencing PA (i.e., positively and negatively) participation in African Americans [10, 12–14], most studies have focused on young adults, and those exploring PA in older AAW have not included women with different levels of PA [12,13,15]. Furthermore, previous studies have depended primarily on interview-based (i.e., focus group or individual interview) methods for data collection. In recent years, investigators have begun to advocate for the adoption of more evocative research methods, not as replacement, but to be used alongside interviews and focus groups. Photo-elicitation is a participatory research technique that invites participants to take photographs of salient features in their lives that are both personally meaningful and possess significant explanatory power. This research technique is based on the simple idea of inserting a photograph into a research interview [16]. It is believed that participants using cameras may better articulate the reality of their lives by focusing on a large array of individual, family, and community resources and needs [17]. Photo-elicitation has been successfully employed in studies addressing eating behavior [18], and in a few PA studies exploring attitudes, beliefs, and preferences around PA topics in older AAW [19], and in the Latino population [20]. For instance, Sebastião et al. [19] successfully employed photo-elicitation in a study exploring PA perceptions among insufficiently active (IA) older AAW. The authors observed that IA women had a vague and non-specific concept of PA. In addition, several personal and environmental facilitators and barriers were reported by the participants. However, to the best of our knowledge, such an approach has yet to be used to explore possible differences in perceptions, barriers and facilitators for PA in groups of regularly active (RA) and IA older AAW.

Ten years ago, a study [21] found that predictors for exercise adherence among younger individuals do not always apply for older adults. Similar concerns have been raised for factors affecting PA in different racial groups. Racially-specific barriers and facilitators that characterize older AAW’s PA beliefs and preferences have not been studied in depth. Exploring such issues is both important and timely given the low rates of PA participation in this population [7,22].

In the present study we employed a mixed-method approach to explore perceptions of PA among RA and IA older AAW and the factors that positively or negatively contribute to their decision to be physically active. This study adopted a participatory research technique known as photo elicitation [16] as a research tool to be used alongside in-depth semi-structured interviews and the objective assessment of PA. The objective assessment of PA was important because it provided valid and reliable data regarding PA, and enabled us to identify two groups that differed with respect to their PA status.

From a theoretical perspective, this study was grounded in the social ecological model of health behavior [23]. Ecological models (i.e., multi-level models) are frequently used to help explain complex behaviors such as PA, and these models are thought to have greater explanatory power than more simplistic single-level approaches. The social ecological model of health behavior suggests that individual behavior does not occur in isolation but emerges from a complex multi-level interaction between the individual, the environment, and the community. Due to the unique attributes of the African American population being studied, we decided to augment the social ecological model of health behavior with theoretical perspectives found in the health disparity literature that have been used to explain the higher burden of chronic diseases in African Americans [24–27]. Health disparities among African Americans are pervasive and numerous factors (i.e., financial issues, the legacy of slavery, segregation and discrimination, and distrust towards biomedical and public health agencies) have been used to try to explain the antecedents of these inequities.

## Materials and Methods

### Study design

A cross-sectional mixed-methods design employing a participatory research technique was adopted. The underlying rationale and the design of the study was driven by the social ecological model of health behavior [23] and was suplemmented by the exploration of theoretical perspectives drawn from the health disparity literature [24–27] (i.e., legacy of slavery, segregation, racism, lack of regular institutional resources, stress, residencial segregation).

### Sample and setting

Twenty older AAW aged 60 to 80 years of age living in Urbana-Champaign, IL were selected for the study. Flyers posted in locations known to have a high flux of African Americans (i.e., local health care and community centers, churches, some food stores), engagement of community leaders, and snowball sampling techniques were used as a recruitment strategy. To be included, participants had to self-report as African Americans aged 60 years and over, walk independently, and be able to answer questions coherently.

### Data collection

Data were collected in three stages by the first author who is of African descent and has a PhD in Cultural and Interpretative Kinesiology and has previous experience in conducting interviews and quantitative measurements of PA in the older adult population. First, participants completed a questionnaire to provide information on age, height, weight, years of education, income level, marital status, self-reported health, number of diagnosed diseases, medication taken, and health insurance status. Second, participants wore an accelerometer at the hip (Actigraph GT3x plus) for 7 consecutive days for objective assessment of PA. Participants were asked to wear the device for at least 10 hours each day. Additionally, participants were instructed to maintain their normal routine.

Along with wearing the accelerometer, participants were given a disposable camera to capture images of their socio-cultural environment. Participants were trained on how to use the camera, which included a practice session to familiarize them with its use. Ethical issues related to the use of cameras in public were discussed, including the potential loss of privacy to the participants, friends, and family, as well as safety issues related to taking photographs in public. Participants were asked to take both positive and negative pictures of resources for engaging in PA, with special attention paid to factors that enable or make it difficult to be physically active. Instructions asked participants to take photos of (a) physically active things that they do; (b) meaningful places they go—indoors and outdoors—that are related to PA and; (c) people they interact with on a daily basis. Furthermore, participants were instructed to include both week and weekend days in their pictures. Along with the verbal instructions mentioned above, participants also received written instructions with the prompts about the pictures. This participatory research approach is known as photo elicitation [16]. After completion, accelerometers and cameras were retrieved for PA data analysis and photo development.

Semi-structured in-depth interviews were employed in combination with the photos to discuss perceptions of PA. The interviews were conducted in a quiet room at the University of Illinois where this study was based. Each participant took an average of 25 photos. Before the interview began, all photos taken by the participant were displayed on a table. Participants were asked to take a look at the set of photos and to choose the four photos that they considered to be the most meaningful to them and that they wished to discuss. The investigator conducting the interview selected two additional photos. Similar approach for photo selection has been previously employed in photo-elicitation research [20]. Although six photos may seem to be a small number from the 25 photos possible, for many participants several photos often depicted the same or similar themes (e.g., church, park, neighborhood). For most participants, the selection of six images was sufficient to adequately represent the diversity of pictures captured. In three instances participants asked to discuss images beyond the four they were initially invited to select. In these cases the participants were allowed to choose and discuss an additional two photographs. The selected photos served as discussion topics for the interviews. The interviewer explored in detail how RA and IA participants viewed PA and PA opportunities in their local environment, and probed about factors that posed as barriers or facilitators for PA. Interviews were conducted in English and lasted about 60 to 75 minutes. During the interview, participants were initially asked questions about the photos and their significance (i.e., What is the image? What is happening in the image? Why is the image important? What resources for PA are shown in the image? Does the picture relate to your personal decision to be active or inactive? Why?).

These initial questions were followed by the semi-structured interview questions that were developed based upon the theoretical frameworks underpinning the study (i.e., social ecological model of health behavior and the health disparity literature). The interview guide was developed by the three researchers involved, two of whom were highly experienced in qualitative studies and in research into underserved populations. At the end of each interview participants were invited to take one last look to the remainder of the photos not included in the initial discussion and allowed to talk about anything that was not previously covered in the interview. The interviews were audio-recorded using a digital recorder, transcribed verbatim to a word document and later uploaded to NVIVO10 [28] software for data management and analysis. Before analysis, the research team validated the transcribed documents by checking them against the audio-record files. The study was not designed to generate separate and discrete findings from the photo elicitation and structured interview segments of the study. Rather, the goal was to utilize both sources of information to develop a generalized impression of factors influencing perceptions and attitudes about PA in older AAW. Thus, the information collected through the pictures and the in-depth interviews were compiled together and presented as one.

### Data analysis

Sociodemographic, biomedical and PA data were analyzed using mean, standard deviation and percentage. The average minutes per week of moderate to vigorous physical activity (MVPA), and minutes per day of accelerometer wear-time were compared between RA and IA groups using a *t* test for independent samples. The number of valid days using the accelerometer was compared using the Mann-Whitney *U test*. For analysis, a significance level of 5% was adopted. This procedure was employed to ensure we were analyzing groups with distinct PA levels.

Accelerometer data were analyzed using ActiLife 6.0 software employing the Freedson et al. adult cut-score equations [29]. The current PA recommendations of 150 minutes per week of MVPA was adopted as the cut point to classify the participants as RA or IA [30], but without considering bouts of 10 minutes or more.

Qualitative data were analyzed using descriptive thematic analysis based on realist and semantic analysis and adopting the 6 phases described by Braun and Clarke [31]. Thematic analysis is an approach for identifying, analyzing, and reporting patterns/themes within data. It organizes and describes the information in rich detail. When all the interviews were completed, two native English speakers with experience in transcribing interviews were hired to transcribe all interviews. Transcriptions were checked for accuracy by the investigators, and analyzed. Initially, each of the three researchers involved in the study separately coded and analyzed the data using NVIVO 10 software [28] to look for themes. Following this process, the researchers compared codes and themes for concordance, retaining only the themes that reached an agreement. When negative cases arose, the researchers discussed each case, using them as an opportunity to further refine each theme. Credibility of the data was examined employing prolonged engagement and investigator triangulation [32].

### Ethics statement

The Institutional Review Board of the University of Illinois approved the research protocols (IRB#: 13309) and a signed informed consent was obtained from all participants before data collection.

## Results

Accelerometer data analysis showed that 9 out of 20 women were RA (i.e., achieved the current PA recommendations of 150 minutes per week of MVPA). The average minutes per week spend in MVPA of the RA women was 270.6 ± 79.9 minutes per week, and 111.9 ± 30.7 minutes for the IA group. The difference was found to be statistically significant (t (18) = - 6.0863; p<0.001). The number of valid days wearing the accelerometer (5.8 vs 5.5; p = 0.58) and accelerometer wear-time (722.4 vs 680.3; p = 0.15) did not differ between groups.

RA and IA groups had similar socio-demographic and health characteristics. Detailed characteristics of the participants are given in Table 1 for both groups.

**Table 1:** General characteristics of the participants.

	Total (n = 20)	Regularly Active (n = 9)	Insufficiently Active (n = 11)
**Age (mean, SD)**	68.3 (6.16)	66.11 (6.39)	70.09 (5.61)
**Education (n, %)**			
High School	5 (25)	2 (22.22)	3 (27.27)
Some College	8 (40)	5 (55.56)	3 (27.27)
College or more	7 (35)	2 (22.22)	5 (45.45)
**Years living in town (mean, SD)**	48.15 (20.97)	43.33 (22.70)	52.09 (16.63)
**Marital Status (n, %)**			
Single	1 (5)	1 (11.11)	—-
Married	7 (35)	3 (33.33)	4 (36.36)
Divorced/Separated	7 (35)	4 (44.44)	3 (27.27)
Widowed	5 (25)	1 (11.11)	4 (36.36)
**Practice of Religion**			
Yes	18 (90)	9 (100)	9 (81.81)
**Children (n, %)**			
Yes	18 (90)	8 (88.89)	10 (90.9)
**Living Status (n, %)**			
Alone	3 (15)	3 (33.33)	—-
Someone live with me	17 (85)	6 (66.67)	11 (100)
**Type of income (n, %)**			
Self-employment	6 (30)	4 (44.44)	2 (18.18)
Government	10 (50)	2 (22.22)	8 (72.72)
Retirement pension	11 (55)	4 (44.44)	7 (63.63)
**Income (n, %)**			
$ 5,000 - $ 9,999	1 (5)	1 (11.11)	—-
$ 15,000 - $ 19,999	3 (15)	3 (33.33)	—-
$ 20,000 - $ 29,999	7 (35)	—-	7 (63.64)
$ 30,000 - $ 39,999	3 (15)	2 (22.22)	1 (9.09)
$ 40,000 - $ 49,999	1 (5)	—-	1 (9.09)
$ 50,000 and over	5 (25)	3 (33.33)	2 (18.18)
**Self-reported health (n, %)**			
Good	9 (45)	5 (55.56)	7 (63.64)
Very good	11 (55)	4 (44.44)	4 (36.36)
**Improving health though exercise, balance diet, and stop smoking? (n, %)**			
Yes	20 (100)	9 (100)	11 (100)
**Chronic Disease (n, %)**			
0	4 (20)	3 (33.33)	1 (9.09)
1–2	11 (55)	5 (55.56)	6 (54.54)
≥ 3	5 (25)	1 (11.11)	4 (36.36)
**Medication (n, %)**			
0	5 (25)	4 (44.44)	1 (9.1)
1–2	9 (45)	3 (33.33)	6 (54.54)
≥ 3	6 (30)	2 (22.22)	4 (36.36)
**Health Insurance (n, %)**			
Yes	19 (95)	8 (88.9)	11 (100)
**BMI (Kg/m** ^**2**^ **; mean, SD)**	30.65 (3.94)	29.23 (4.14)	31.82 (3.53)
**Difficulties in ADL or IADL (n, %)**			
Yes^a^	2 (10)	—-	2 (18.18)
No	18 (90)	—-	9 (81.81)
**Depressive symptoms (n, %)**			
Yes^b^	2 (10)	1 (11.11)	1 (9.09)
**Fall in the past 12 months (n, %)**			
Yes	5 (25)	2 (22.22)	3 (27.27)
**Afraid of Falling (n, %)**			
Yes	7 (35)	4 (44.44)	3 (27.27)

BMI: Body Mass Index–calculated by the weight divided by the height squared

^a^ Participant reported some difficulty in walking.

^b^ depressive symptoms was based on the positive answer for the questions “Lately, have you felt down, depressed, or hopeless?; and “Lately, have you felt little interest or pleasure in doing things?” for identifying possible traces of depression.

### Knowledge and perceptions of physical activity

Both RA and IA women held two major beliefs about the nature of PA. Some women’s understanding of PA was consistent with a broadly defined construct that goes beyond traditional exercise routines, and is consistent with the concept of leading a physically active lifestyle. However, others expressed beliefs that PA and exercise are synonymous and can be used interchangeably. The following are illustrative examples from the structured interviews;

- **“**I think physical activity is anything that gets you up and moving. So it could be walking, bicycling, skating, any type of sports that you participate in… I don’t think it’s something that has to be that formal…” (66—Inactive)
- “…wake up in the morning with a floor exercise, followed by my stationary bike. And I would do that twice a day, in the mornings, in the evenings… that was probably the most that I’ve done”. (65—Active)

Regardless PA status, many women expressed a lack of understanding about the amount of PA people in their age need in order to achieve health benefits. However, others had a better understanding of recommended frequency and duration of PA. Virtually all participants lacked an appreciation for the role that intensity of exercise plays in achieving reliable health benefits. Some women expressed a belief that women in their age should be as active as their body allowed them to be, suggesting that PA frequency, duration, and intensity should be established by individual preference rather than by an externally established norms and guidelines.

- “… I just don’t remember. It is a lot of things… but I’ve heard some of the statistics, I’ve heard, but I can’t quote them to you…” (68—Inactive)
- “…at least three times a week doing something… At least two hours… I think a person should actively do something at least three times a week, because one is not enough” (66—Active)
- “I do as much as I can, as the body will take… So I don’t have a standard that I say, “OK, I need to do an hour’s worth of this, or I need to do that…” (68—Inactive)

Participants were able to describe many of the benefits of regular PA. Regardless of PA status, women discussed the impact of PA on both physical and mental health, quality of life and overall well-being, and the importance of PA for maintaining independence. Both active and sedentary women also discussed the importance of healthy eating as an important component of a healthy lifestyle.

- “… physical activity helps reduction of diabetes…weight gain… I think a lot of those chronic diseases… a lot of chronic conditions come from, I think, from a lack of exercise…”(66—Inactive)
- “… I want to live a long, and good, happy, prosperous life…. it helps my mental state of mind, to just–to walk, it helps me. I feel, like if I’m depressed or feeling low, I go on a walk and I forget all about it” (62—Active)
- “… as long as I keep moving and doing something in order to keep my muscles strong so that I don’t have to have help getting up and down… Cause I’m getting older and older and I can tell the body changes”. (78—Inactive)
- “… I don’t overeat and I try to get into some physical activity… I think that makes for healthy living… That to me is if you have that balance between those very key components of your life…” (60—Active)

### Factors influencing physical activity participation

#### Physical health and psychological and emotional health

Participants reported that health issues often hinder them from being physically active. Although health concerns were reported by both RA and IA women, IA participants were more likely to report physical health problems and the RA women were more likely to identify psychological and emotional health concerns.

- “… right now I have an issue with lower back pain… even though I try to do things, it’s still hindering me, and I’m not as active as I could be … since the injury, it’s really decreased.” (62—Inactive)
- “… We have a lot of challenges that a lot of other communities don’t have. There’s a lot of trauma in the African American community… it’s like an injury that never heals kinda trauma… mentally you’re drained from the challenges that you have to deal with, just by virtue of being African American, you’re tired. You have limited mental ability to deal with everything else…” (60—Active)

#### Financial problems

Participants in both groups mentioned that African Americans often live on a fixed income that often limits exercise and PA choices;

- “… when it comes down to fitness, African Americans I just think are traditionally hard working people … when you look at some of the gyms and some of the fitness centers their memberships were just astronomic.” (60—Active)

#### Family demands

Some participants, regardless of PA status, expressed that their role as caregivers in their families prevented them from being more physically active.

- “… [She] has cancer, and every time she has an appointment I go down to talk with her and the doctors…. That’s the big thing why I am not in physical activity classes…” (74—Inactive)

#### Friends and family

For some RA and IA women, friends and family enabled them to be more active.

- “… She’s my walking buddy, so we walk inside because she doesn’t like to walk outside… I used to have another walking buddy, and we used to walk outside, but then my other walking buddy couldn’t walk outside anymore, so I started walking with her…” (62—Active)
- “My daughter lives in Rantoul… I go up to help motivate her, as well as myself, to walk this track. We don’t run it, we walk it. And we just have mother and daughter time… at least once a week…” (62—Inactive)

#### Unsafe neighborhood

RA and IA women alike often perceived their neighborhoods as unsafe for PA, mentioning problems related to bad sidewalks and poor lighting at night, and also by discussing their fear of crime.

- “You see this here [talking about a cracked sidewalk]. I almost tripped and fell on my face. My friend … actually did that and she had to go to the doctor … That is like about 2 inches up and if you are walking and you are not looking, anybody … could fall and hurt themselves” (72- Active)
- “… I don't like to go [walk in the evening, night]; I don’t like to go to places that I can’t see… It’s not too much light over in our area because we’re not paying to the city lights up there”. (73—Inactive)
- “… the least I like about it, is at night. And that doesn't necessarily mean this in my neighborhood but you hear some shooting.” (66—Inactive)

#### Green areas and parks

Despite the problems reported by the participants in their neighborhoods, both RA and IA women identified green areas and parks as facilitators for PA:

- “I had a park around the corner … is the park where a lot of walkers walk around the park. I don’t know if it’s estimated to be a couple miles, I don’t know, but it’s there … it is conducive to a lot of physical activity….” (60—Active).

#### Churches and senior centers

Regardless of PA status, participants discussed the importance of local churches and senior centers as a resource for PA. Participants mentioned health promotion initiatives developed by their churches that encouraged participation in PA. Others identified senior centers as a place for socialization and also as a facilitator for PA.

- “… My pastor is very much into health and wellness… They have a get up and move… He gets up out and we maybe walk around the park and he’s always challenging us… we’ll take those long walks … our church is very health conscious.” (72—Active)
- “… It’s our dance at Douglas Center… she is the dance instructor, and it’s about six of us that participate in the program, and it’s a good form of exercise…” (72—Inactive)

#### Fitness and recreation centers

RA and IA participants mentioned that having access to fitness and recreation centers served as a facilitator for PA. Some RA and IA participants discussed the activities and equipment that are available in those locations.

- “…I have a picture here of the Planet Fitness center … I enjoy, it’s a lot of people, lot of various activities going on in there, and different treadmills, and all kinds of exercising equipment. So it’s a great place to go to get exercise.” (80—Active)

Table 2 summarizes the barriers and facilitators reported by the participants.

**Table 2:** Factors influencing physical activity participation in regularly active and insufficiently active older African American women.

Domain	Barriers	Facilitators
**Individual**	Physical health	
	Psychological and emotional health	
	Financial problems	
**Interpersonal**	Family demands	Friends and family
**Environment**	Bad sidewalks	Green areas and parks
	Poor lighting	
	Neighborhood crime	
**Community**		Churches and senior centers
		Fitness and recreation centers

## Discussion

To our knowledge this study is one of the first studies to conduct an in-depth investigation of PA and factors influencing PA in older AAW employing a participatory approach (i.e., photo-elicitation). The findings of this study may help in the development of more culturally sensitive interventions and strategies to promote PA in this particularly vulnerable population. The study adds to the literature by exploring factors impacting PA in an under-represented, at risk group using an innovative research design including the objective measurement of PA and the use of photo-elicitation. Findings from our study suggest that: (a) some factors positively and negatively impacting PA are similar among RA and IA older AAW; (b) older AAW lack knowledge about the PA intensity necessary to achieve health benefits.

We observed that, regardless PA status, individuals think about PA in several different ways. Some women believe that PA encompasses a wide range of activities, including both traditional exercise, as well as, “lifestyle activities” such as gardening, housekeeping, and walking the dog. This broad interpretation of PA is consistent with the definition most frequently used in contemporary public health initiatives [5,30]. However, other individuals appear to be using a more traditional interpretation of PA that is synonymous with “exercise”, i.e., an activity that occurs at a specific place, usually for a specific duration and intensity, and often with the specific purpose of increasing physical fitness [5,33]. This finding of different interpretations for the “meaning” of PA is consistent with the findings of several studies examining perceptions, attitudes and factors influencing PA and exercise in both Whites and African Americans [12,34,35].

We observed that while some RA and IA women have a good understanding of the amount of PA needed in terms of frequency and duration, others in both groups had no idea of how much PA individuals in their age should perform. Although many participants mentioned frequency and duration of PA, none had an appreciation of the intensity of PA needed to obtain health benefits. This is unfortunate because intensity is a critically important component of PA for achieving health benefits. It is important to note that our findings about intensity may reflect the way we inquired about participant’s knowledge. Participants were asked how much PA should women in their age do and if they ever had heard about any government recommendations regarding PA. Follow up questions explored such topics as how many days, for how long, and at what intensity? However, we never explicitly probed participants using the words “moderate” or “vigorous intensity”. Many public health initiatives have targeted completely sedentary individuals in an effort to motivate them to build some level of activity into their everyday lives and settings (e.g., family, workplace, communities and clinical settings) [36]. For the most part, such initiatives fail to adequately stress the importance of PA intensity. There is solid evidence in the PA and exercise literature that not only frequency and duration but also intensity of exercise are necessary elements in order to achieve health benefits [1,5]. Some participants in this study expressed the belief that women in their age group should be as active as their bodies allowed them to be. This is an interesting finding and it is consistent with parts of the 2008 PA guidelines for Americans [4]. Older adults aged 65 and over unable to achieve the 150 minutes per week because of a chronic condition, should be as active as they can be, matching their activities to their fitness level. This government recommendation is consistent with the clinical characteristics of our sample, where 55% of the women in this study reported 1 or 2 chronic diseases; and 25% reported having 3 or more chronic diseases. Our findings suggest that many older AAW may have difficulty in setting realistic PA goals that are consistent with evidence-based guidelines. Wilcox et al., [12] observed that participants in their study held the opinion that frequency, duration and intensity should be chosen by the individual and not guided by an external criterion. Together, these findings suggest that there is a need to more effectively disseminate information about evidence-based guidelines and recommendations for PA. Individuals should be aware of the amount of PA needed per week in terms of frequency, duration and intensity in order to achieve substantial health and quality of life improvements.

Factors influencing PA participation in this study were generally consistent with the various levels of the social ecological model of health behavior [23]. Our findings corroborate the results of prior studies with similar purposes [10,11,14,37]. For instance, studies using focus groups to explore barriers and facilitators for PA have observed similar barriers and facilitators for PA participation as the present study [10,11]. Although in many studies health issues were reported as both a barrier and facilitator for PA [13,14,38], in the present study, health concerns emerged mainly as a barrier despite the fact some participants acknowledged an association between health and PA. Women in this study understood that health benefits accrue from PA, however, they seldom identified poor health as a factor that motivated them to be more active. On the other hand, others mentioned that they refrain from being more active because of their health conditions. This suggests that barriers and facilitators for PA may vary from person to person and that not all health messages are equally effective in all circumstances. This highlights the importance of conducting additional research in different AA communities in order to develop an in-depth knowledge about the community beliefs and preferences prior to implementing behavioral change programs. Leavy and Aberg [38] qualitatively explored perceptions of PA in older Swedish and Irish adults. They found that physically active and inactive participants reporting chronic diseases (e.g., arthritis, heart disease) have a distinct perception about health problems. Active participants viewed health problems as motivators for PA whereas inactive participants viewed health problems mainly as barriers to be more active. It is important to note that barriers and facilitators to PA are extremely challenging to compare between studies. While some studies have used a quantitative approach based on questionnaires or a list of statements [39–41], others have adopted qualitative approaches employing different methods for data collection, i.e. focus groups, interviews [10,11,14]. For instance, Siddiqi et al., [14] in their review identified at least four different types of experimental design, with focus groups being the most prevalent among older adults. Therefore, differences found between our study and others could be partially explained by the study design adopted, methods of data collection employed and sample characteristics.

Participants in both RA and IA group expressed the perception that living in an unsafe neighborhood (i.e., bad sidewalks, poor lighting, and dangerous neighborhood) was a barrier to PA. In fact, information provided by the cities of Urbana-Champaign, suggests that the concerns raised about neighborhood safety are often grounded in reality. For instance, a number of neighborhoods have inadequate or non-existent lighting. Furthermore, the violent crime rate in Champaign is more than twice the average observed in the State of Illinois.

This study also found that economic issues and psychosocial stress due to life conditions may be hindering older AAW from being more active. It is important to note that economic issues have been constantly reported by African Americans and seldom mentioned by other racial groups as barriers to PA [11,14]. Psychosocial stress (e.g., racism, discrimination) has been used by sociologists primarily to understand disparities in diseases and conditions and access to and type of care received [28]. Psychosocial stress in the context of PA participation has been much less frequently studied. Edwards and Cunningham [42] examined how opportunities for PA and community racism are associated with decreased engagement in PA. The authors observed that when community racism was low, respondents found ways to be active; however, when community racism was high, perceived lack of opportunities significantly obstructed PA engagement. It is not clear whether factors such as discrimination and life history alter how the Social Ecological Model applies in different ethnic and/or racial groups. Future research should explore whether the interactions among levels of the social ecological model are impacted by the disproportional burdens faced by many African Americans that may deter them from engaging in PA.

The findings of the present study constitute important information for public health practice and for health professionals aiming to implement PA programs for older AAW living in the community. In view of the findings, action will be needed from different sectors if we are to increase PA levels among older African Americans with special attention being paid to providing access to no or low cost opportunities to be active in safe environments. Williams and Jackson [43] note that health disparities are embedded in a larger historical, geographic, sociocultural, economic, and political context. Therefore, changes in a broad range of public policies are needed to effectively address these systemic and ongoing disparities.

Our findings provide insight with respect to some of the ways RA and IA older AAW are similar. Socio-demographic variables, health characteristics, or factors influencing PA cannot be used to explain why some women are active and others are sedentary. However, objective PA data collected shows that some participants are achieving the PA recommendations and some are not. Although we do not have clear evidence with regard to why this is the case, a study conducted by Edwards and Cunningham [42] on perceived racism and level of PA offers a partial explanation. The IA group in our study may perceive higher levels of racism in the community compared to the RA group and may not be fully aware of the whole range of possibilities for being active. Additionally, RA women may have found ways to keep motivated and maintain enjoyment in being physically active [44]. Developing strategies to buffer the impact of racism/discrimination on healthy lifestyle choices may be one way to increase PA level in this group and should be further explored.

This study benefited from the use of both the objective measurement of PA and the use of photo-elicitation. Distinct from other methods of data collection (e.g., interview or focus groups), photo-elicitation helped in breaching the communication impasse, bridging geographical and cultural gaps between investigator and participants, and making participants feel more aware of and responsible for their role and importance in the study. The combination of both qualitative and quantitative approaches is a promising research design for in-depth understanding of behavior, attitudes, and perceptions. Although important, our findings must be interpreted with care due to the following limitations. Qualitative research has its limitations, including the inability to generalize findings to a reference population, (i.e., the general population of older AAW living in the United States). Our study was based on women living in a small town, relatively well educated, with a reasonable annual income. Accordingly, caution is warranted when generalizing/transferring the outcomes to the broader population of older AAW. An additional limitation of our study was that data were only collected during one season of the year (winter/spring/summer/fall), it is possible that participant’s perceptions and values regarding PA may fluctuate from season to season. It would be helpful for a study to explore how seasonal variation impacts these perceptions. Finally, we did not require that MVPA occur in bouts of at least 10 minutes when determining whether or not participants were meeting the PA recommendations. Rather, we employed a more nuanced approach by counting every minute in MVPA, which is consistent with current public health messages used to increase PA at the population level. However, it is important to note that differences in PA level have been reported when comparing MVPA data between “bout dependent” and “bout independent” approaches [7, 45].

## Supporting Information

S1 File Interview guide used during the interviews. (PDF)
