# Supplementary material for: An In-Depth Examination of Perceptions of Physical Activity in Regularly Active and Insufficiently Active Older African American Women: A Participatory Approach
Source: PLoS One. 2015 Nov 10;10(11):e0142703. doi: 10.1371/journal.pone.0142703 (PMC4640571; doi:10.1371/journal.pone.0142703)
Supplement: S1 File — (PDF) [file pone.0142703.s001.pdf]

Individual interview questions

An in-depth examination of perceptions of physical activity in regularly active and insufficiently active older African American women: A participatory approach

A. Individual level

Broader question: What does living healthy mean to you?

- What does physical activity means to you? Is physical activity the same as exercise?
- How important is physical activity to you? To your health?
- How much physical activity should a woman your age do?

B. Relationship level (family; social network, etc.)

Broader question: Is there anything special or different about health in African American families?

- Tell me about your role in your family?
- Do you try to help members of your family stay healthy? How?
- Is physical activity something common among your family members?
- In what ways do you think your family, friends encourage or discourage you to be more active?

C. Community level

Broader question: How do you feel about your neighborhood?

- Tell me about your neighborhood.
- Is it easy to be physically active in your neighborhood? What makes it easy? What makes it hard?
- Are they any groups or programs in your neighborhood that promote health and physical activity?
- Has anyone ever told you about how much physical activity you should do? Have you ever discussed this with your doctor or nurse?

D. Elements of Health Disparities

Broader question: There are several studies suggesting that African Americans are more vulnerable to diseases because of many different factors, such as lack of public resources

nearby their neighborhood, higher level of psychosocial stress throughout life, economic issues, etc. Could you comment on this based on your life experiences?

- Could you tell me if any of the mentioned factors **[repeat to the participant the factors]** affect your willingness to be physically active? How?

- During the summer time, for example, you decide to go to a park or any other green area for a walk. How far or how close do you have to go? How would you get there? Bus, car, bicycle, walking? Would you call a friend or relative to join you? Why, or why not?

- Now think about the winter time. What are some options you have available to you, except at your house, to exercise; to walk; to meet with your friends or relatives?

- Tell me about your close friends. Are they physically active in your opinion? Would be okay for you to join them? Why, or why not?

- There are several government health recommendations that are based on scientific and medical research. What are your feelings about medical research?

- Many experts say that physical activity can be used to treat different diseases. What do you think about that?

- The current government guidelines recommend that older adults should do at least 2 hours and 30 minutes of physical activity per week. How do you feel about that?
